# Sarcopenia as a poor prognostic indicator for renal cell carcinoma patients undergoing nephrectomy in China: A multicenter study

**DOI:** 10.1002/ctm2.270

**Published:** 2021-01-01

**Authors:** Weipu Mao, Keyi Wang, Hui Zhang, Haowen Lu, Si Sun, Changxiu Tian, Zonglin Wu, Jianping Wu, Bin Xu, Huae Xu, Bo Peng, Ming Chen

**Affiliations:** ^1^ Department of Urology Affiliated Zhongda Hospital of Southeast University Nanjing China; ^2^ Surgical Research Center Institute of Urology, Southeast University Medical School Nanjing China; ^3^ Department of Urology, Nanjing Lishui District People's Hospital, Zhongda Hospital Lishui Branch Southeast University Nanjing China; ^4^ Department of Urology, Shanghai Tenth People's Hospital, School of Medicine Tongji University Shanghai China; ^5^ Department of Anesthesiology, Shanghai Tenth People's Hospital, School of Medicine Tongji University Shanghai China; ^6^ Department of Urology Shidong Hospital of Yangpu District Shanghai China; ^7^ Department of Pharmaceutics, School of Pharmacy Nanjing Medical University Nanjing China

Dear Editor,

We conducted a multicenter clinical study to investigate the effect of sarcopenia on survival of Chinese population after nephrectomy for renal cell carcinoma (RCC). Lumbar skeletal muscle index (SMI) and total psoas index (TPI) measured by preoperative computed tomography were used to assess sarcopenia. And we found that sarcopenia was a poor prognostic indicator of overall survival (OS) and cancer‐specific survival (CSS).

RCC is one of the most common malignancies in the urinary system and also the most common renal malignant tumor originating from the kidney, accounting for 80‐85% in renal malignant tumors and 2‐3% in systemic malignancies.^1^ For patients with localized RCC, radical or partial nephrectomy is the most common treatment. For patients with advanced metastatic RCC, comprehensive treatments such as molecular replacement therapy and immunotherapy are widely used.^2^

Sarcopenia, also known as skeletal muscle loss, is a progressive and extensive skeletal muscle disease characterized by reduced skeletal muscle mass and decreased muscle function.^3^ Many current studies have confirmed that the occurrence of sarcopenia is closely associated with the treatment and prognosis of many resectable malignant tumors.^4^ Sarcopenia can reduce the treatment tolerance of tumor patients, increase the toxic reaction of antineoplastic drugs, prolong the length of hospital stay, and increase postoperative complications, which is the index of poor prognosis of tumor patients after operation.^5^

For patients with RCC, the prognostic value of sarcopenia is still controversial. Sharma et al^6^ and Fukushima et al^7^ found that sarcopenia is an OS‐related factor for patients with metastatic RCC (mRCC) who have received cytoreductive nephrectomy or cytokine therapy and targeted agents. However, Peyton et al^8^ found that sarcopenia was not associated with OS in advanced RCC patients who have received radical nephrectomy. Furthermore, there are no studies to verify the prognostic value of sarcopenia in Chinese RCC patients.

We conducted a large retrospective multicenter study among the Chinese population from three hospitals between January 2014 and December 2019 to evaluate the effect of sarcopenia on OS and CSS in patients with RCC undergoing nephrectomy. Sarcopenia was assessed with lumbar SMI and TPI measured by computed tomography within 1 month before surgery. About TPI, the total psoas area (TPA, mm^2^) on both sides of the L3 axial plane was assessed, and then the TPI (TPI = TPA/(height (m) × height (m))) was normalized to the patient's height. About SMI, the skeletal muscle area (SMA, cm^2^) is the total muscle area of the psoas, paraspinal, internal oblique, external oblique, rectus abdominis, and transversus abdominis muscles on both sides (Figures 1A‐1C), and then SMI (SMI = SMA/(height (m) × height (m))) was normalized to height. Patients with TPI < 545mm^2^/m^2^ in male or TPI < 385mm^2^/m^2^ in female can be diagnosed as sarcopenia.^9^ Patients in female with SMI < 41cm^2^/m^2^ or in male with SMI < 43cm^2^/m^2^ and body mass index (BMI) < 25 kg/m^2^ or with SMI < 53cm^2^/m^2^ and BMI ≥25 kg/m^2^ can be diagnosed as sarcopenia.^10^

**FIGURE 1:**
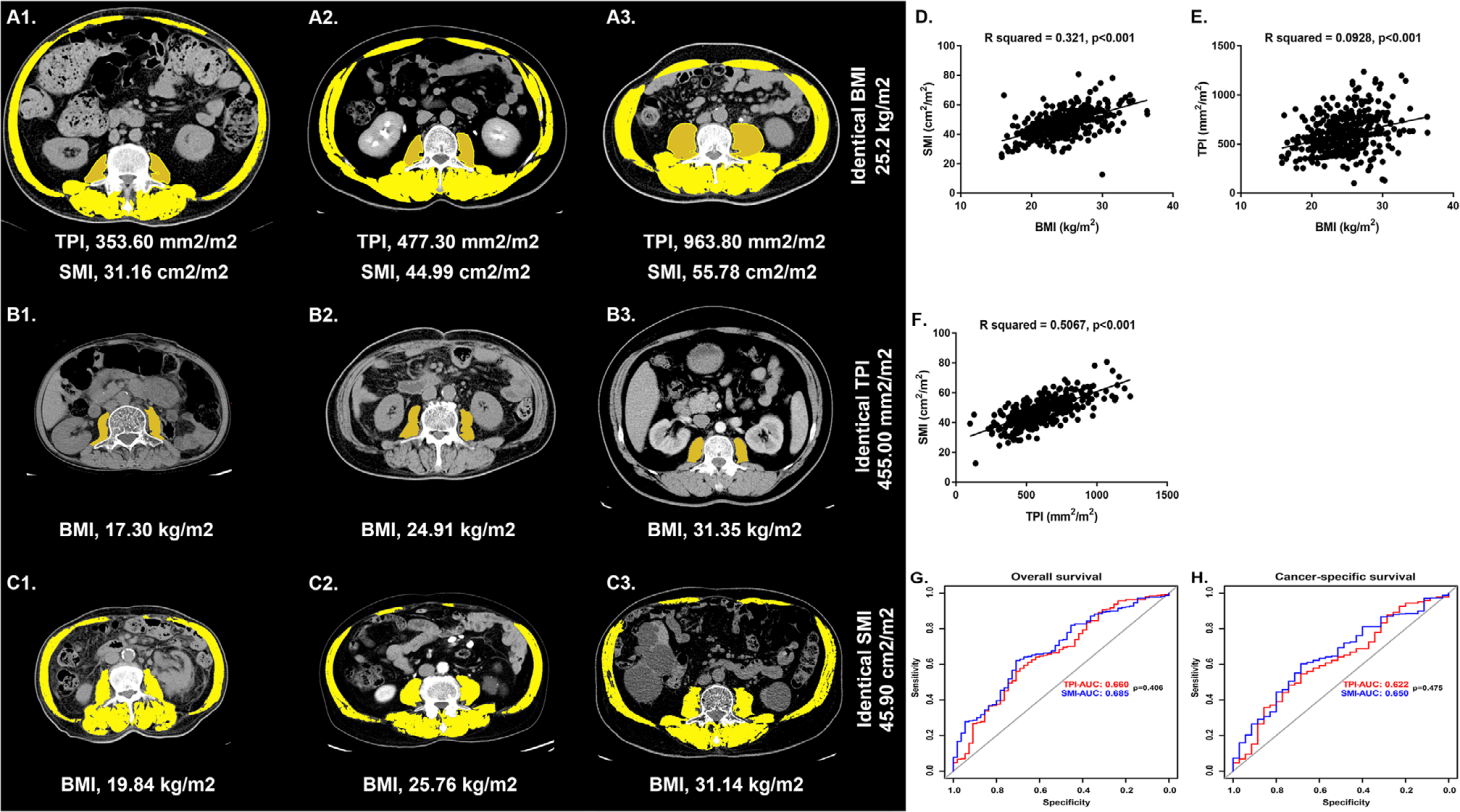
The axial CT images of the third lumbar region, the correlation between TPI, SMI, and BMI, and the ROC curve of TPI and SMI to evaluate the predictive value of OS and CSS. A, The images of male patients with the same BMI (25.2 kg/m^2^) and different TPI and SMI. B, The images of male patients with the same TPI (455.00 mm^2^/m^2^) and different BMI. C, The images of male patients with the same SMI (45.90 cm^2^/m^2^) and different BMI. D, SMI and BMI correlation, Person correction on coefficient, R = 0.321, *P* < .001. E, TPI and BMI correlation, Person correction on coefficient, R = 0.0928, *P* < .001. F, SMI and TPI correlation, Person correction on coefficient, R = 0.5067, *P* < .001. G, ROC curve of OS. H, ROC curve of CSS. Abbreviations: BMI, body mass index; CSS, cancer‐specific survival; OS, overall survival; ROC, receiver operating characteristic; SMI, skeletal muscle index; TPI, total psoas index

When the TPI was used as an assessment criterion for sarcopenia, a total of 97 patients (21.9%) could be assessed as sarcopenia (Table 1). When the SMI was used as an evaluation criterion for sarcopenia, 157 patients (35.4%) were assessed as sarcopenia. In the entire cohort, the mean age of patients was 58.02 years, and the gender was predominantly male (66.8%). The proportion of female patients and over 65 years patients in the sarcopenia group was higher than that in nonsarcopenia group, but the difference observed when TPI was used as an assessment criterion was not statistically significant (gender, *P* = .493; age, *P *= .237). Patients with sarcopenia had older age, lower BMI, and shorter survival time than nonsarcopenia patients. Due to the lower BMI of sarcopenia patients, we examined the relationship among TPI, SMI, and BMI. And then we found a significant correlation between SMI and BMI (R square = 0.321, *P* < .001; Figure 1D), TPI and BMI (R square = 0.0928, *P* < .001; Figure 1E), SMI and TPI (R square = 0.5067, *P* < .001; Figure 1F).

**TABLE 1:** Baseline characteristics with comparison between sarcopenia and nonsarcopenia patients when using TPI or SMI as an assessment tool

		TPI			SMI		
	All patients	Nonsarcopenic	Sarcopenic		Nonsarcopenic	Sarcopenic	
Characteristic	No. (%)	No. (%)	No. (%)	*P* value	No. (%)	No. (%)	*P* value
Total patients	443	346 (78.1)	97 (21.9)		286 (64.6)	157 (35.4)	
Age, years, mean ± SD	58.02 ± 12.44	57.54 ± 12.31	59.72 ± 12.82	.127	57.26 ± 11.84	59.39 ± 13.40	.084
Age categorized, years				.237			.032
≤65	318 (71.8)	253 (73.1)	65 (67.0)		215 (75.2)	103 (65.6)	
>65	125 (28.2)	93 (26.9)	32 (33.0)		71 (24.8)	54 (34.4)	
Gender				.493			.012
Male	296 (66.8)	234 (67.6)	62 (63.9)		203 (71.0)	93 (59.2)	
Female	147 (33.2)	112 (32.4)	35 (36.1)		93 (29.0)	64 (40.8)	
BMI, kg/m^2^, mean ± SD	24.60 ± 3.55	25.13 ± 3.36	22.71 ± 3.57	<.001	25.30 ± 3.38	23.34 ± 3.51	<.001
BMI categorized, kg/m^2^				<.001			.682
<25	251 (56.7)	180 (52.0)	71 (73.2)		160 (55.9)	91 (58.0)	
≥25	192 (43.3)	166 (48.0)	26 (26.8)		126 (44.1)	66 (42.0)	
Hypertension				.036			.027
No	251 (56.7)	187 (54.0)	64 (66.0)		151 (52.8)	100 (63.7)	
Yes	192 (43.3)	159 (46.0)	33 (34.0)		135 (47.2)	57 (36.3)	
Diabetes				.628			.392
No	372 (84.0)	289 (83.5)	83 (85.6)		237 (82.9)	135 (86.0)	
Yes	71 (16.0)	57 (16.5)	14 (14.4)		49 (17.1)	22 (14.0)	
Cardiovascular diseases				.134			.773
No	392 (88.5)	302 (87.3)	90 (92.8)		254 (88.8)	138 (87.9)	
Yes	51 (11.5)	44 (12.7)	7 (7.2)		32 (11.2)	19 (12.1)	
Smoking				.062			.973
No	370 (83.5)	295 (85.3)	75 (77.3)		239 (83.6)	131 (83.4)	
Yes	73 (16.5)	51 (14.7)	22 (22.7)		47 (16.4)	26 (16.6)	
Surgery type				.012			<.001
Partial nephrectomy	268 (60.5)	220 (63.6)	48 (49.5)		191 (66.8)	77 (49.0)	
Radical nephrectomy	175 (39.5)	126 (36.4)	49 (50.5)		95 (33.2)	80 (51.0)	
Laterality				.246			.205
Left	224 (50.6)	166 (48.0)	53 (54.6)		151 (52.8)	73 (46.5)	
Right	219 (49.4)	180 (52.0)	44 (45.4)		135 (47.2)	84 (53.5)	
Histological type				.303			.520
Clear cell carcinoma	351 (79.2)	275 (79.5)	76 (78.4)		229 (80.1)	122 (77.7)	
Papillary cell carcinoma	23 (5.2)	19 (5.5)	4 (4.1)		17 (5.9)	6 (3.8)	
Chromogenic carcinoma	19 (4.3)	17 (4.9)	2 (2.1)		11 (3.8)	8 (5.1)	
Others	50 (11.3)	35 (10.1)	15 (15.5)		29 (10.1)	21 (13.4)	
AJCC stage				.003			.070
I	329 (74.3)	271 (78.3)	58 (59.8)		223 (78.0)	106 (67.5)	
II	26 (5.9)	16 (4.6)	10 (10.3)		15 (5.2)	11 (7.0)	
III	60 (13.5)	40 (11.6)	20 (20.6)		35 (12.2)	25 (15.9)	
IV	28 (6.3)	19 (5.5)	9 (9.3)		13 (4.5)	15 (9.6)	
T‐stage				.001			.159
T1	336 (75.8)	277 (80.1)	59 (60.8)		226 (79.0)	110 (70.1)	
T2	30 (6.8)	20 (5.8)	10 (10.3)		18 (6.3)	12 (7.6)	
T3	66 (14.9)	41 (11.8)	25 (25.8)		37 (12.9)	29 (18.5)	
T4	11 (2.5)	8 (2.3)	3 (3.1)		5 (1.7)	6 (3.8)	
N‐stage				.538			.415
N0	425 (95.9)	333 (96.2)	92 (94.8)		276 (96.5)	149 (94.9)	
N1	18 (4.1)	13 (3.8)	5 (5.2)		10 (3.5)	8 (5.1)	
M‐stage				.107			.109
M0	424 (95.7)	334 (96.5)	90 (92.8)		277 (96.9)	147 (93.6)	
M1	19 (4.3)	12 (3.5)	7 (7.2)		9 (3.1)	10 (6.4)	
Fuhrman grade				.397			.146
I	74 (16.7)	61 (17.6)	13 (13.4)		52 (18.2)	22 (14.0)	
II	276 (62.3)	216 (62.4)	60 (61.9)		183 (64.0)	93 (59.2)	
III	83 (18.7)	63 (18.2)	20 (20.6)		46 (16.1)	37 (23.6)	
IV	10 (2.3)	6 (1.7)	4 (4.1)		5 (1.7)	5 (3.2)	
Urea nitrogen, mmol/L	6.45 ± 4.41	6.57 ± 4.83	6.03 ± 2.32	.285	6.50 ± 5.11	6.36 ± 2.71	.741
Creatinine, μmol/L	111.80 ± 88.22	115.02 ± 97.83	100.30 ± 35.67	.147	114.61 ± 101.89	106.68 ± 55.18	.366
Uric acid, μmol/L	278.41 ± 102.62	277.49 ± 101.24	281.71 ± 107.88	.721	283.16 ± 100.99	269.76 ± 105.30	.189
TPI, mm^2^/m^2^, mean ± SD	595.90 ± 179.85	645.63 ± 164.90	418.52 ± 102.51	<.001	639.77 ± 177.60	515.99 ± 155.15	<.001
SMI, cm^2^/m^2^, mean ± SD	47.38 ± 8.41	49.23 ± 7.83	40.79 ± 7.01	<.001	50.83 ± 6.84	41.09 ± 7.29	<.001
Survival time (months)	32.88 ± 19.52	33.60 ± 19.40	30.31 ± 19.48	.143	33.22 ± 19.92	32.25 ± 18.81	.620

**Abbreviations**: AJCC, American Joint Committee on Cancer; BMI, body mass index; SD, standard deviation; SMI, skeletal muscle index; TPI, total psoas index.

Smoothing splines curves showed that both TPI and SMI could reduce the risk of OS (Figures 2A and 2C) and CSS (Figures 2B and 2D) as their values increased. As there are differences in gender, ethnic, and physical between Eastern and Western countries, we redefined the thresholds of TPI and SMI for the diagnosis of sarcopenia in Chinese patients with RCC (Table S1). In the total population, the thresholds for TPI and SMI were 574.1mm^2^/m^2^ and 47.5cm^2^/m^2^, respectively (Figures 2A‐2D).

**FIGURE 2:**
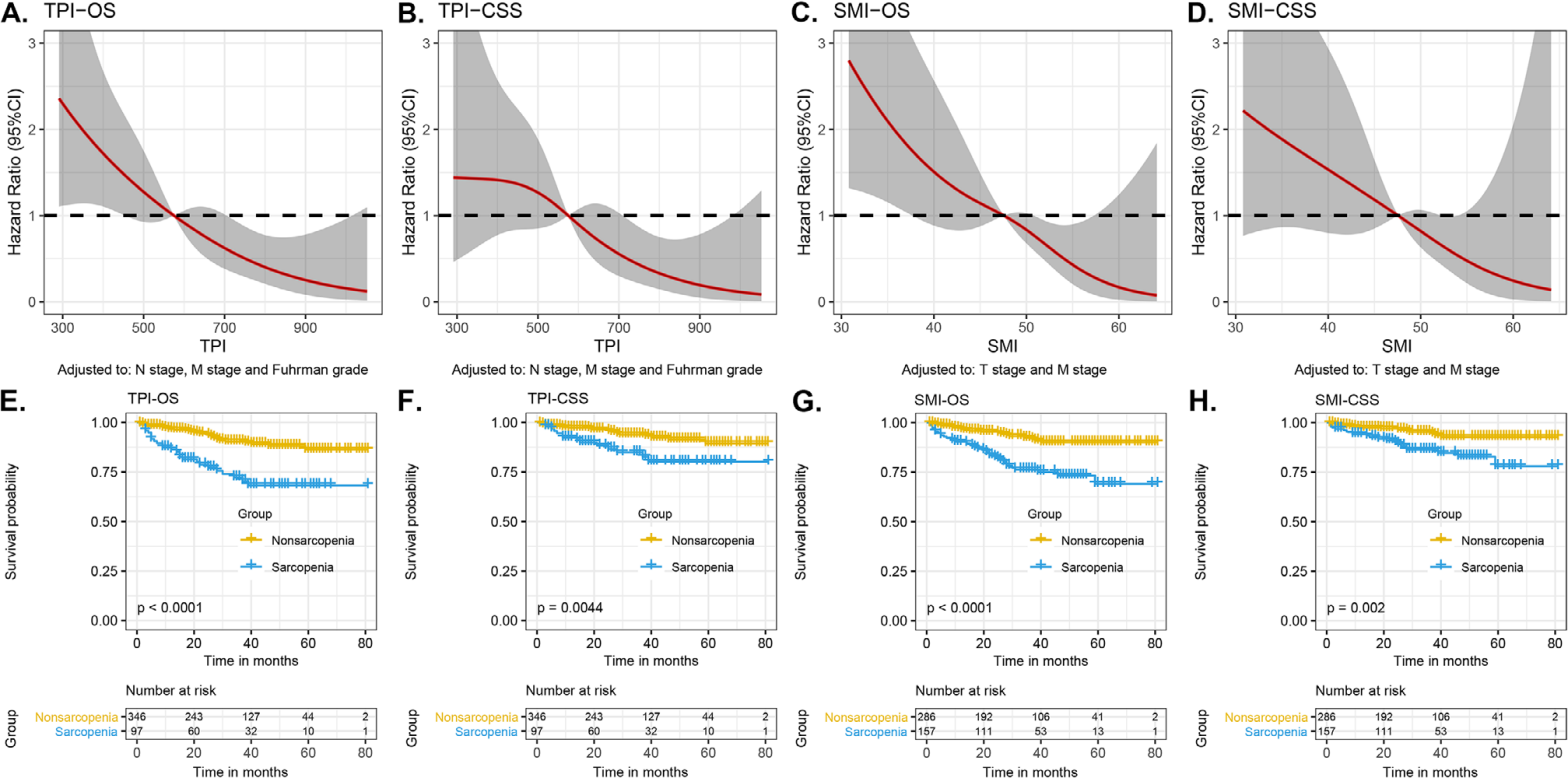
Relationship between TPI, SMI, and OS, and CSS. A, Graphical illustrations of TPI and OS. B, Graphical illustrations of TPI and CSS. C, Graphical illustrations of SMI and OS. D, Graphical illustrations of SMI and CSS. E, Kaplan‐Meier curve for OS in sarcopenia and nonsarcopenia as indexed by TPI. F, Kaplan‐Meier curve for CSS in sarcopenia and nonsarcopenia as indexed by TPI. G, Kaplan‐Meier curve for OS in sarcopenia and nonsarcopenia as indexed by SMI. H, Kaplan‐Meier curve for CSS in sarcopenia and nonsarcopenia as indexed by SMI. Abbreviations: CSS, cancer‐specific survival; OS, overall survival; SMI, skeletal muscle index; TPI, total psoas index.

The median follow‐up time in the whole cohort was 32.0 months, and the median follow‐up time of survivors was 34.5 months. As of the end of the follow‐up, 55 patients (12.4%) were dead and 35 (7.9%) of whom died of RCC. The 5‐year OS (TPI: 68.3% vs 86.2%, *P* < .001, Figure 1E; SMI: 69.0% vs 90.0%, *P* < .001, Figure 1G) and 5‐year CSS (TPI: 80.2% vs 89.5%, *P* = .004, Figure 1F; SMI: 77.9% vs 92.7%, *P* = .002, Figure 1H) were significantly lower in sarcopenia patients compared with nonsarcopenia patients.

We used Cox proportional hazard regression models to determine whether sarcopenia is a factor affecting the survival of patients with RCC. Tables S2 and S3 showed the hazard risks to OS and CSS for patients with sarcopenia estimated by TPI or SMI. In univariate Cox analysis, sarcopenia was a risk factor for OS and CSS in patients with RCC. In addition, sarcopenia was an independent risk factor to OS and CSS in patients with RCC, whether in the base model, core model, or extended model (TPI‐OS: HR = 2.745; 95% confidence intervals (CI) 1.581‐4.755; *P* < .001; SMI‐OS: hazard ratios (HR) = 2.884; 95% CI 1.657‐5.018; *P* < .001; TPI‐CSS: HR = 2.181; 95% CI 1.076‐4.460; *P* = .031; SMI‐CSS: HR = 2.578; 95% CI 1.284‐5.150; *P* = .009). Moreover, ROC curves showed that there was no statistical difference between the TPI and SMI measures in assessing sarcopenia for predicting OS (TPI: AUC = .660, SMI: AUC = .685; *P* = .406) and CSS (TPI: AUC = .622, SMI: AUC = .650; *P* = .475) (Figures 1G and 1H).

To our knowledge, this is the largest and also the first multicenter study on the effect of sarcopenia to the prognosis of Chinese nephrectomy patients. And it shows that sarcopenia is a factor which can affect the poor prognosis of OS and CSS in nephrectomy patients.

## CONFLICT OF INTEREST

The authors declare that there is no conflict of interest that could be perceived as prejudicing the impartiality of the research reported.

## ETHICS APPROVAL AND CONSENT TO PARTICIPATE

The methodology of this study followed the criteria outlined in the Declaration of Helsinki (as revised in 2013) and was ethically approved by the Ethics Committees and Institutional Review Boards of all participating institutions.

## Supporting information

Supporting Information

## Data Availability

The datasets used and analyzed during the current study are available from the corresponding author on reasonable request.
