# Supplementary material for: Sarcopenia as a poor prognostic indicator for renal cell carcinoma patients undergoing nephrectomy in China: A multicenter study
Source: Clin Transl Med. 2021 Jan 1;11(1):e270. doi: 10.1002/ctm2.270 (PMC7775986; doi:10.1002/ctm2.270)
Supplement: Supplementary file 1 — Supporting Information [file CTM2-11-e270-s001.docx]

**Table S1**. Total psoas index (TPI) and skeletal muscle index (SMI) threshold values.

| BMI (kg/m2) | Current study cohort | | Fearon et al. | | | Current study cohort | | | Martin et al. | |
| --- | --- | --- | --- | --- | --- | --- | --- | --- | --- | --- |
|  | TPI (mm2/m2) | | TPI (mm2/m2) | | | SMI (cm2/m2) | | | SMI (cm2/m2) | |
|  | Male | Female | | Male | Female | | Male | Female | Male | Female |
| All | 624.4 | 446.0 | | 545 | 385 | | - | - | - | - |
| < 25 | - | - | | - | - | | 47.0 | 40.0 | 43 | 41 |
| ≥ 25 | - | - | | - | - | | 54.5 | 44.2 | 53 | 41 |

**Abbreviations:** BMI, Body mass index; TPI, total psoas index; SMI, skeletal muscle index.

**Table S2**. Hazard risk for overall survival (OS) for patients with sarcopenia estimated by total psoas index (TPI) or skeletal muscle index (SMI).

| Models | TPI | | SMI | |
| --- | --- | --- | --- | --- |
|  | HR (95% CI) | P value | HR (95% CI) | P value |
| Univariate analysis | 3.234 (1.907-5.485) | <.001 | 3.218 (1.863-5.560) | <.001 |
| Basic model | 3.223 (1.900-5.498) | <.001 | 3.211 (1.853-5.572) | <.001 |
| Core model | 2.753 (1.591-4.760) | <.001 | 2.878 (1.654-5.014) | <.001 |
| Extended model | 2.745 (1.581-4.755) | <.001 | 2.884 (1.657-5.018) | <.001 |

**Abbreviations:** OS, Overall survival; TPI, total psoas index; SMI, skeletal muscle index; HR, hazard ratio; CI, confidence interval; AJCC, American Joint Committee on Cancer.

Basic model: age, gender, hypertension, diabetes, cardiovascular diseases and smoking; Core model: basic model plus surgery type, laterality, histological type, AJCC stage, T stage, N stage, M stage, and Fuhrman grade; Extended model: core model plus BMI.

**Table S3.** Hazard risk for cancer-specific survival (CSS) for patients with sarcopenia estimated by total psoas index (TPI) or skeletal muscle index (SMI).

| Models | TPI | | SMI | |
| --- | --- | --- | --- | --- |
|  | HR (95% CI) | P value | HR (95% CI) | P value |
| Univariate analysis | 2.577 (1.315-5.052) | .006 | 2.778 (1.417-5.446) | .003 |
| Basic model | 2.562 (1.301-5.058) | .006 | 2.771 (1.408-5.461) | .003 |
| Core model | 2.196 (1.080-4.461) | .029 | 2.582 (1.290-5.152) | .007 |
| Extended model | 2.181 (1.076-4.460) | .030 | 2.578 (1.284-5.150) | .009 |

**Abbreviations:** CSS, cancer-specific survival; TPI, total psoas index; SMI, skeletal muscle index; HR, hazard ratio; CI, confidence interval; AJCC, American Joint Committee on Cancer.

Basic model: age, gender, hypertension, diabetes, cardiovascular diseases and smoking; Core model: basic model plus surgery type, laterality, histological type, AJCC stage, T stage, N stage, M stage, and Fuhrman grade; Extended model: core model plus BMI.
